# Pediatric Unspecified Headache in the Emergency Department: Can We Do Better?

**DOI:** 10.5811/westjem.62197

**Published:** 2026-10-18

**Authors:** Sarah R. Martin, Theodore W. Heyming, Eric C. Sun, Jenny Zhu, Harshul Zaveri, Zeev N. Kain

**Affiliations:** * University of California, Irvine School of Medicine, Department of Anesthesiology & Perioperative Care, Irvine, California; † University of California, Irvine, Center on Stress & Health, Irvine, California; ‡ Rady Children’s Health, Orange County, Emergency Medicine, Orange, California; § University of California, Irvine, Department of Emergency Medicine, Orange, California; ¶ Rady Children’s Health, Orange County, Department of Neurology, Orange, California; || Stanford University, Perioperative and Pain Medicine, Department of Anesthesiology, Stanford, California; # University of California, Irvine School of Medicine, Irvine, California; ** Rady Children’s Health, Orange County, Department of Pediatrics, Orange, California; †† Yale University Child Study Center, New Haven, Connecticut

## Abstract

**Introduction:**

Unspecified headache is a common discharge diagnosis in the pediatric emergency department (ED). While diagnostic uncertainty and limited access to specialty evaluation may contribute to repeat ED visits in this population, longitudinal utilization data are limited. Study objectives were both to determine the incidence of repeat headache-related ED visits in children discharged from the ED with unspecified headache and to identify predictors of ED return (eg, prior headache-related ED or neurology utilization).

**Methods:**

This preliminary, prospective longitudinal cohort study included 76 children (ages 8–17 years) discharged from the ED with an unspecified headache diagnosis. We assessed headache-related ED and outpatient neurology visits both six months after the index visit (ie, ED enrollment) and, retrospectively, 12 months before the index visit. The sample included children insured through a Medi-Cal–based Physician-Hospital Consortium, who receive care exclusively within an integrated healthcare network, and other children in the hospital network, allowing capture of all ED and clinic utilization data. Our primary outcome was incidence of headache-related ED utilization postindex visit. Pain characteristics were self-reported during the index visit. Chi-square, Mann-Whitney U, and logistic regression analyses examined factors associated with incidence of headache-related ED returns.

**Results:**

Nearly 12% (*n* = 9) of children returned to the ED for a headache 6 months following the index visit. Across the 18-month study, 22.4% had at least one headache-related ED visit outside the index visit, 64.7% (*n* = 11) of whom had ≥2 headache-related ED encounters. A prior headache-related ED visit or prior neurology visit was significantly associated with returning to the ED after the index visit (20.0% vs 3.0%; risk difference = 17.0%; 95% CI, 0.9%–48.0%; *P* = .03, and 37.5% vs 1.5%; risk difference = 28.7%; 95% CI, 3.2%–60.9%; *P* = .02, respectively). Children with more than two headache-related ED visits during the 18-month study reported higher pain intensity (7.00 [SD, 1.38] vs 5.79 [SD, 2.09]; mean difference = −1.21; 95% CI, −2.20 to −0.22; *P* = .02). In logistic regression analyses, prior neurology utilization was the only variable significantly associated with headache-related ED return (OR, 15.31; 95% CI, 1.08–216.34; *P* = .04).

**Conclusion:**

A considerable proportion of children discharged with unspecified headache returned to the ED for headache. Emergency department and neurology utilization and pain intensity were linked to return ED visits. Larger, longitudinal studies are needed to further clarify these findings.

## INTRODUCTION

Headache is a common pediatric complaint, affecting up to 60% of children with as many as 30% experiencing recurrent episodes,^1^–^3^ and is associated with significant pain burden, contributing to substantial healthcare utilization and costs.^4^,^5^ Headache complaints account for approximately 3% of pediatric emergency department (ED) visits, with rates rising nearly three times faster than overall pediatric ED utilization.^6^–^8^ Most cases are attributed to non-life-threatening conditions (85–98%),^7^ most commonly primary or unspecified headache disorders (50–66%).^6^,^7^,^9^,^10^

Emergency clinicians face challenges in differentiating between primary headache subtypes due to time constraints and barriers to neurology consultation. Consequently, many children are discharged with a nonspecific or unspecified headache diagnosis, limiting follow-up guidance and contributing to repeat ED utilization.^7^,^10^,^11^ Currently, there is a paucity of data on recurrent ED utilization in this population. Existing research, which is limited to brief follow-up periods of up to 1 month, report return rates of 4–8%.^12^–^14^ We submit the need for longer-term ED and outpatient neurology utilization data for unspecified headache diagnosis and identification of children at risk for recurrent ED visits.

This preliminary investigation characterized ED utilization patterns among children diagnosed with unspecified headache, examining incidence of ED use in the 12 months preceding and 6 months following the study index visit, as well as subsequent outpatient neurology encounters.

## METHODS

### Design and Participants

This cohort study employed a prospective, longitudinal design combined with a retrospective data component. Participant enrollment was conducted within a pediatric ED in the southwestern United States from November 2022–June 2024. Data were collected prospectively following ED enrollment (ie, index visit) and retrospectively to capture utilization prior to the index visit. Participants included a convenience sample of children 8–17 years old who were discharged from the ED with an unspecified headache diagnosis (International Classification of Diseases, Tenth Revision R51.9). Attending emergency physicians assigned discharge diagnoses. Diagnosis eligibility was then reviewed by study investigators, including a pediatric emergency physician. Patients were excluded if they had an Emergency Severity Index (ESI) score of 1 or were not fluent in English or Spanish.

### Measures

#### Demographics

Children and caregivers reported demographics including sex, ethnicity, and race.

#### Clinical Acuity

Emergency department ESI scores (level 1 = most critically ill to level 5 = least critically ill) were obtained from the electronic health record (EHR).^15^

**Population Health Research Capsule**

What do we already know about this issue?

*Unspecified headache is a common diagnosis in the pediatric emergency department (ED) and diagnostic uncertainty may contribute to repeat visits.*

What was the research question?

*What is the incidence of repeat headache-related ED visits in children discharged with unspecified headache?*

What was the major finding of the study?

*Across 18 months, 22% had >2 headache ED visits; prior neurology care predicted 6-month ED return (OR, 15.31 [1.08–216.34], P =. 04).*

How does this improve population health?

*Reporting headache-related ED utilization will inform future research and interventions to improve pediatric headache care in the ED.*

#### Length of Stay

The ED length of stay (hours) was obtained from the EHR.

#### Pain intensity and duration

Children self-reported pain intensity via a numeric rating scale ranging from 0 (“no pain”) to 10 (“worst pain possible”)^16^ and pain duration categorized as pain ≤1 month, >1 month, and pain ≥3 months.^17^

#### ED and Neurology Utilization

Emergency department visits with a headache chief complaint and outpatient neurology encounters 12 months before and 6. months after the index visit (ie, ED enrollment visit) were extracted from medical records. We used the incidence of headache-related ED and neurology encounters in analyses. The sample included children who were uninsured, had other insurance, or were insured through the Medi-Cal–based Physician-Hospital Consortium (PHC) and received care exclusively within an integrated healthcare network where all encounters were captured. Utilization data were identifiable for PHC and non-PHC participants receiving care within the healthcare network, which includes two EDs and primary and specialty care clinics across four counties.

### Procedures

Ethical approval for study procedures was obtained from the institutional review board (IRB) (#220577). Research personnel identified patients who met chief complaint, ESI, and age eligibility through the ED tracking board and approached patients to conduct screening and informed consent. Enrolled participants then completed measures on REDCap, a secure data collection tool. Study investigators then reviewed ED discharge diagnoses. Participants discharged with an unspecified headache diagnosis were included in the current analyses. Electronic health record and utilization data were extracted using advanced electronic/automated Structured Query Language queries. Extraction adhered to Worster et al^18^ case-selection criteria, variable definitions, hypothesis blinding, medical record identification, and IRB approval criteria.

### Statistical Analyses

Analyses were performed using IBM SPSS Statistics, version 29.0 (IBM Corp, Armonk, NY). We dichotomized ED and neurology utilization to indicate encounter occurrence. Before primary analyses, preliminary chi-squared tests revealed no significant differences in prior ED visits (*P* = .30) or ED returns (*P* = .49) between PHC-enrolled patients (*n* = 34–44.7%) and non-PHC members (*n* = 42, 55.3%). Therefore, the combined sample was used for primary analyses. Chi-square and Mann-Whitney U tests examined associations of demographics, pain, and prior utilization with incidence of headache-related ED returns. Effects sizes were reported as phi φ (small =. 0.1, medium = 0.3, large =. 0.5) or Cohen *d* (small = 0.2, medium = 0.5, large = 0.8). A McNemar test examined changes from pre- to postindex neurology visits. A multivariable logistic regression examined independent associations with likelihood of headache-related ED return after the index visit.

Independent variable selection was informed by headache literature identifying demographic (age, sex, ethnicity) and pain characteristics (intensity and duration) associated with headache outcomes,^19–22^ and current preliminary results suggesting that prior headache-related ED and neurology utilization is associated with ED returns. Exploratory analyses examined factors associated with headache-related ED utilization over the 18-month study period. Given the preliminary nature of this study and convenience sample, an a priori power analysis was not conducted. Post hoc sensitivity analyses indicated that the sample (*N* = 76) was powered to detect large effects (OR ≥ 6.05) at 80% power, α = .05.

## RESULTS

Of the 174 patients who presented to the ED with headache, 76 were discharged with an unspecified headache diagnosis and were included in the current analyses. Excluded diagnoses included migraine (*n* = 17); secondary headache (infection *n* = 35; injury *n* = 14; homeostasis (*n* = 5); cranial/vascular (*n* = 4); structural disorder (*n* = 5); psychiatric (*n* = 2); and other/non-headache (*n* = 16). Participant characteristics are presented in Table 1.

### Emergency Department and Outpatient Neurology Utilization (Table 1)

Across the 18-month study period (12 months pre-ED index and 6 months post-ED index), 22.4% of participants had at least one additional headache-related ED visit outside the index visit, approximately 65% of whom had two or more headache-related ED encounters. In the 12 months prior to the ED index visit, 13.2% of participants had a headache-related ED visit. In the 6 months postindex visit, 11.8% of participants returned to the ED for a headache; of these, seven received an unspecified headache diagnosis at subsequent visits, one a migraine diagnosis, and one left without being seen. Prior to the ED index visit, 10.5% of participants had an outpatient neurology encounter for headache symptoms. Neurology utilization significantly increased to 27.6% following the index visit (McNemar test *P* = .002).

### Factors Associated with Returning to the Emergency Department for Headache

Bivariate analyses indicated that the incidence of a prior headache-related ED visit was significantly associated with returning to the ED within one month (*P* = .03) but not within six months of the index visit (*P* = .10). Prior neurology and postindex neurology utilization were significantly associated with returning to the ED within six months (*P* = .02; *P* = .04, respectively). Pain intensity was moderately higher in children who returned to the ED for headache (*d* = .62, *P* = .11). Table 2 presents bivariate results.

Multivariable logistic regression results indicated that prior neurology utilization was the only variable significantly associated with incidence of headache-related ED return within six months (*P* = .04; Table 3).

In exploratory analyses, children with 2 or more headache-related ED visits reported significantly higher pain intensity than those with only the index visit (mean difference = −1.21; 95% CI, −2.20 to −0.22; *P* = .02). Pain duration was not significantly associated with having ≥2 ED visits, but small effects emerged for pain >1 month (φ = 0.23, *P* = .08) and chronic pain (φ = 0.20, *P* = .15). Children with pain >1 month or chronic pain were more likely to have two or more ED visits (25.0%) than those without (8.5%; risk difference = 16.9%; 95% CI, −1.2% to 36.0%, and 9.8%; risk difference = 15.2%; 95% CI, −3.0% to 36.0%).

## DISCUSSION

In this preliminary investigation, during an 18-month period, over 20% of children discharged from an ED with unspecific headache diagnosis had at least one additional headache-related ED visit. This exceeds previously reported return rates of 4–8% from studies using shorter follow-up periods,^12^–^14^ underscoring the healthcare burden in this population and need for future research to capture longer care trajectories and identify intervention targets to improve headache management and reduce recurrent ED use. The current higher return rates may reflect our unique Medi-Cal–based PHC, which enabled collection of comprehensive healthcare utilization data. This contrasts with previous studies that may have underreported utilization when children sought care outside the original hospital system.

In multivariable regression analyses, prior outpatient neurology utilization was the only variable significantly associated with returning to the ED for headache within six months. Age, gender, ethnicity, pain symptoms, and prior headache-related ED visits were not. While this finding may reflect greater underlying clinical complexity among children previously seen by neurology, it may also indicate gaps in treatment effectiveness, care coordination, or access to follow-up. Prior consensus recommendations have identified fragmented care pathways and variability in headache management across settings as contributors to recurrent ED utilization.^23^ Clinically, prior neurology utilization may serve as a marker for children who could benefit from enhanced ED discharge planning or closer outpatient follow-up; however, given the preliminary nature of this study, future research incorporating additional clinical and treatment data is needed to clarify the mechanisms underlying this association.

Although pain symptoms were not significantly associated with postindex ED returns, children with two or more headache-related ED visits over the 18-month study reported significantly higher pain intensity. Small, non-significant effects were observed for pain duration with 25% of children reporting pain for over one month had two or more ED visits—nearly triple the rate of those without recurrent pain. These findings must be interpreted with consideration of a type II error. Collectively, trends suggest that headache pain severity and chronicity may contribute to ED use and a need for larger longitudinal studies to clarify how pain characteristics and treatment influence headache-related ED utilization.

## LIMITATIONS

Several limitations should be considered. The modest sample size may have limited statistical power, increasing likelihood of a type II error. Larger samples and studies designed to examine headache-related treatment trajectories and response over time are needed to develop multivariable risk models to inform clinical decision-making. This study relied on ED discharge diagnoses assigned during standard clinical care which may limit interpretation of diagnostic specificity or introduce diagnostic misclassification. The single-site design may limit generalizability to other ED populations. For non-PHC participants, utilization data were restricted to encounters within the PHC network, potentially missing external encounters. Future studies incorporating longitudinal patient-reported care data may better characterize utilization.

## CONCLUSION

Overall, findings suggest that children discharged from the ED with unspecified headache have high rates of ED utilization extending beyond the brief follow-up windows used in previous research. The mechanisms underlying the association between prior neurology utilization and increased risk for ED return warrants further investigation. Results from this preliminary study highlight the need for comprehensive, longitudinal research that includes patient- and system-level variables (eg, access to headache care, institutional referral practices) to better understand multilevel factors driving ED reliance and clinical outcomes in this understudied population.

## Figures and Tables

**Table 1:** Demographic and clinical characteristics, and incidence of emergency department (ED) and neurology utilization in a sample of children who presented to an ED with headache and received a diagnosis of unspecified headache.

Variable	*n* (%)	Mean (SD)
Child age (years)		13.89 (2.68)
Child sex		
Female	46 (68.7)	
Male	a21 (31.3)	
Ethnicity		
Latino	57 (86.4)	
Non-Latino	9 (13.6)	
Race		
African American, Black	1 (1.8)	
Asian, Pacific Islander	1 (1.8)	
Native American, Alaskan	1 (1.8)	
White	28 (49.1)	
Multiracial, Other	26 (45.6)	
Caregiver language		
English	46 (64.8)	
Spanish	25 (35.2)	
Emergency Severity Index		
2	2 (2.7)	
3	68 (90.7)	
4	4 (6.7)	
Length of stay (hours)		5:36 (2:20)
Pain intensity		6.20 (1.95)
Pain duration		
< 1 month	37 (56.9)	
≥ 1 month to < 3 months	4 (6.2)	
≥ 3 months	24 (36.9)	
Utilization and Time Period Variables	Sample *n* (%)	
12 Months Prior to ED Index Visit		
ED visit for headache	10 (13.2)	
Within 1 month	7 (9.2)	
Within 3 months	8 (10.5)	
Within 6 months	8 (10.5)	
Neurology visit	8 (10.5)	
6 Months Following ED Index Visit		
ED visit for headache	9 (11.8)	
Within 1 month	4 (5.3)	
Within 3 months	6 (7.9)	
Neurology visit	21 (27.6)	
Entire 18-Month Study Period*		
ED Visit for headache		
≥ 1 ED visit for headache	17 (22.4)	
≥ 2 ED visits for headache	11 (14.5)	

* Does not include study index visit.

*ED*, emergency department; *SD*, standard deviation.

**Table 2:** Results of chi-square and mean difference analyses examining associations between demographic, clinical, and prior utilization variables and the incidence of return emergency department visits for headache within 6 months.

Variables	Headache ED Return within 6 months Mean (SD) or *n* (%)		Risk or Mean Difference (95% CI†)	*P*	Effect Size*

	No	Yes			
Utilization					
Prior headache ED visit			9.4% [−15.7–34.5]	.39	.10
No	59 (89.4)	7 (10.6)			
Yes	8 (80.0)	2 (20.0)			
Prior neurology visit			17.0% [0.9–48.0]	.02	.27
No	62 (91.2)	6 (8.8)			
Yes	5 (65.5)	3 (37.5)			
Postneurology visit			16.5% [−3.8–37.0]	.04	.23
No	51 (92.7)	4 (7.3)			
Yes	16 (76.2)	5 (23.8)			
Demographic					
Child age (years)	13.94 (2.75)	13.48 (2.09)	0.46 [−1.44–2.36]	.63	.17
Child sex			5.7% [−14.0–25.0]	.53	.08
Female	39 (84.8)	7 (15.2)			
Male	19 (90.5)	2 (9.5)			
Ethnicity			19.5% [−49.0–10.0]	.09	.20
Latino	51 (89.5)	6 (10.5)			
Non-Latino	7 (88.9)	3 (11.1)			
Race				.95	.11
African American, Black	1 (100)	0 (0)			
Asian, Pacific Islander	1 (100)	0 (0)			
Native American, Alaskan	1 (100)	0 (0)			
White	25 (89.3)	3 (10.7)			
Multiracial, Other	22 (84.6)	4 (15.4)			
Caregiver language			1.0% [−18.0–20.0]	.90	.02
English	40 (87.0)	6 (13.0)			
Spanish	22 (88.0)	3 (12.0)			
Clinical					
ESI				.59	.12
2	2 (100)	0 (0)			
3	59 (86.8)	9 (13.2)			
4	5 (100)	0 (0)			
Length of stay (hours)	5:28 (2:17)	6:35 (2:35)	1:60 [−2:45–0:32]	.18	.48
Pain intensity	6.04 (1.92)	7.22 (1.99)	−1.19 [−2.57–0.20]	.09	.62
Pain duration >1 month			0.8% [−16.0–18.0]	.93	.01
No	32 (86.5)	5 (13.5)			
Yes	24 (87.5)	4 (14.3)			
Pain duration ≥3 months			4.5% [−14.0–22.0]	.61	.06
No	36 (87.8)	5 (12.2)			
Yes	20 (83.3)	4 (16.7)			

* Phi φ reported as effect size for chi-square analyses and Cohen *d* reported for mean difference analyses.

† Risk differences not reported for race and ESI due to extreme sparsity in groups.

*ED*, emergency department; *ESI*, Emergency Severity Index; *SD*, standard deviation.

**Table 3:** Results of a logistic regression analysis examining independent associations between demographic, pain, and prior utilization variables and incidence of returning to the emergency department (ED) for headache within 6 months of the index ED visit.

Variable	*B*	*SE*	Wald *x^2^*	*P*	*OR* (95% CI)
Child age	−0.22	0.19	1.40	.24	0.8 (0.56–1.15)
Child sex (female)	−0.51	1.09	0.22	.64	0.6 (0.07–5.09)
Child ethnicity (Latino)	0.29	1.06	0.07	.79	1.33 (0.17–10.55)
Pain intensity	0.57	0.30	3.48	.06	1.76 (0.97–3.18)
Pain duration (> 1 month)	−0.90	1.02	0.78	.38	0.41 (0.06–2.99)
Prior ED visit for headache (yes)	0.03	1.28	0.00	.98	1.03 (0.08–12.76)
Prior neurology visit (yes)	2.73	1.35	4.08	.04	15.31 (1.08–216.34)

*ED*, emergency department; *OR*, odds ratio; *SE*, standard error.

## References

[1] Chambers CT, Dol J, Tutelman PR (2024). The prevalence of chronic pain in children and adolescents: a systematic review update and meta-analysis. Pain.

[2] Onofri A, Pensato U, Rosignoli C (2023). Primary headache epidemiology in children and adolescents: a systematic review and meta-analysis. J Headache Pain.

[3] Dooley JM, Gordon KE, Wood EP (2005). Self-reported headache frequency in Canadian adolescents: validation and follow-up. Headache.

[4] Bethell C, Kemper KJ, Gombojav N (2013). Complementary and conventional medicine use among youth with recurrent headaches. Pediatrics.

[5] Law EF, Palermo TM, Zhou C (2019). Economic impact of headache and psychiatric comorbidities on healthcare expenditures among children in the United States: a retrospective cross-sectional study. Headache.

[6] Massano D, Julliand S, Kanagarajah L (2014). Headache with focal neurologic signs in children at the emergency department. J Pediatr.

[7] Mourid MR, Oweidat M, Okesanya OJ (2025). Pediatric headache management in the emergency department: a review of challenges and solutions. Curr Treat Options Pediatr.

[8] Perry MC, Yaeger SK, Toto RL (2018). A modern epidemic: increasing pediatric emergency department visits and admissions for headache. Pediatr Neurol.

[9] Raucci U, Della Vecchia N, Ossella C (2019). Management of childhood headache in the emergency department: review of the literature. Front Neurol.

[10] Rossi R, Versace A, Lauria B (2018). Headache in the pediatric emergency department: a 5-year retrospective study. Cephalalgia.

[11] Kellier D, Anto MM, Hall M (2025). Association of race and ethnicity with emergency room rate of migraine diagnosis, testing, and management in children with headache. Neurology.

[12] Hemberg JL, Pomeranz ES, Rogers AJ (2020). Functional impairment predicts outcome of youth with headache in the emergency department. Clin Pediatr (Phila).

[13] Sheridan DC, Dhatt S, Narayan K (2020). Effectiveness of emergency department treatment of pediatric headache and relation to rebound headache. Pediatr Emerg Care.

[14] Costa FM, Ferreira IP, Mascarenhas IF (2020). Diagnosis and treatment of headache in a pediatric emergency department. Pediatr Emerg Care.

[15] Wuerz RC, Milne LW, Eitel DR (2000). Reliability and validity of a new five-level triage instrument. Acad Emerg Med.

[16] Williamson A, Hoggart B (2005). Pain: a review of three commonly used pain rating scales. J Clin Nurs.

[17] Kent ML, Tighe PJ, Belfer I (2017). The ACTTION–APS–AAPM Pain Taxonomy (AAAPT) multidimensional approach to classifying acute pain conditions. J Pain.

[18] Worster A, Bledsoe RD, Cleve P (2005). Reassessing the methods of medical record review studies in emergency medicine research. Ann Emerg Med.

